# TERT promoter mutations are associated with distant metastases in upper tract urothelial carcinomas and serve as urinary biomarkers detected by a sensitive castPCR

**DOI:** 10.18632/oncotarget.2660

**Published:** 2014-12-09

**Authors:** Kun Wang, Tiantian Liu, Nan Ge, Li Liu, Xiaotian Yuan, Jikai Liu, Feng Kong, Chang Wang, Hongbo Ren, Keqiang Yan, Sanyuan Hu, Zhonghua Xu, Magnus Björkholm, Yidong Fan, Shengtian Zhao, Cheng Liu, Dawei Xu

**Affiliations:** ^1^ Department of Urology, Shandong University Qilu Hospital, Jinan, China; ^2^ Department of Medicine, Division of Hematology and Centre for Molecular Medicine, Karolinska University Hospital Solna and Karolinska Institutet, Stockholm, Sweden; ^3^ Department of Pathology, Shandong University School of Medicine, Jinan, China; ^4^ Department of Urology and Central Research Laboratory, Shandong University Second Hospital, Jinan, China; ^5^ Shandong University School of Nursing, Jinan, China; ^6^ Department of General Surgery, Shandong University Qilu Hospital, Jinan, China

**Keywords:** Cancer biomarkers, Promoter mutations, Telomerase, TERT, UTUC

## Abstract

TERT promoter C228T and C250T mutations occur in various malignancies including bladder cancer (BC) and may serve as urinary tumor markers. However, the mutation association with clinical variables in upper tract urothelial carcinomas (UTUCs) is unclear. There is also a lack of sensitive tools to detect the minor mutant TERT promoter in bulk urinary DNA. Here we analyzed 220 UTUC patients [98 with renal pelvic carcinoma (RPC) and 122 with ureter carcinoma (UC)] and developed a Competitive Allele-Specific TaqMan PCR (castPCR) for urinary assay. We identified C228T or C250T mutations in 42 of 98 (43%) RPC and 23 of 122 (19%) UC tumors. Distant metastases were significantly correlated with UTUC patients harboring TERT promoter mutations (*P* = 0.001). C228T were detected in 6/10 and 9/10 of urine samples from patients with mutation-carrying tumors using Sanger sequencing and castPCR, respectively. When urine samples from 70 BC patients were analyzed together, the sensitivity of urinary C228T assay was 89% and 50% for castPCR and Sanger sequencing, respectively (*P* < 0.001). Collectively, TERT promoter mutations occur in UTUCs with a high frequency in RPCs and predict distant metastasis. castPCR assays of the mutation are a useful tool for urine-based diagnostics of urological malignancies.

## INTRODUCTION

The cancer-specific expression of telomerase reverse transcriptase (TERT) and telomerase activation play a pivotal role in malignant transformation and progression, and therefore, the underlying mechanism has been extensively investigated [1–3]. Recently, hotspot mutations in the TERT promoter namely C228T and C250T were identified in various human malignancies including urological tumors and the mutations create *de novo* ETS1 binding motifs, thereby facilitating TERT transcription and telomerase activation in cancer cells [4–16]. Because the mutations are not present in normal cells or tissues, their detection shows a great promise for cancer diagnostics and disease surveillance. Up to 84% of bladder cancer (BC) carry TERT promoter mutations and the mutant sequence is detectable in voided urine from mutation-positive BC patients [7, 8, 11]. Thus, the TERT promoter mutation may serve as a urinary biomarker in BC diagnostics. In addition, the presence of TERT promoter mutations has also been demonstrated to be associated with poor patient outcome in several types of cancer including BC [12, 13, 17, 18]. The accumulated data have collectively suggested that the detection of TERT promoter mutations has important clinical implications.

Upper tract urothelial carcinomas (UTUCs), like BC, are derived from the urothelium or belong to transitional cell carcinomas and consist predominantly of renal pelvic carcinomas (RPCs) and ureter carcinomas (UTs) [19, 20]. Compared to BC, UTUC is less frequent and comprises < 10% of urothelial carcinomas, however, the incidence has increased over the past two decades, and moreover, most UTUCs have become invasive when discovered, mainly due to lack of early clinical symptoms and of useful diagnostic tools [20]. Hence, the identification of reliable bio-markers and development of accurate urine-based diagnostics of UTUCs is a demanding task to improve clinical management and patient outcomes.

Most UTUCs exhibit telomerase activation and express TERT [21], and a few studies based on very limited numbers of UTUC patients also showed the presence of TERT promoter mutations [9, 10, 12]. However, the relationship between TERT mutation status and clinical variables in UTUCs has never been explored. In addition, it remains unclear whether TERT promoter mutations occur in both RPCs and UCs, and whether they are detectable in patients' urine, as seen in BC and can be used as a diagnostic marker for UTUCs. The present study was designed to address these clinically-relevant issues. Moreover, given the published observation that the sensitivity for detection of the mutant TERT promoter in BC urine by Sanger sequencing is low [9, 10], we developed a Competitive Allele-Specific TaqMan PCR (castPCR) [22] (http://tools.lifetechnologies.com/content/sfs/brochures/cms_095916.pdf.) to improve the sensitivity of the patient urine assay.

## RESULTS

### Differential TERT promoter mutation rate between RPCs and UCs

**Figure 1:**
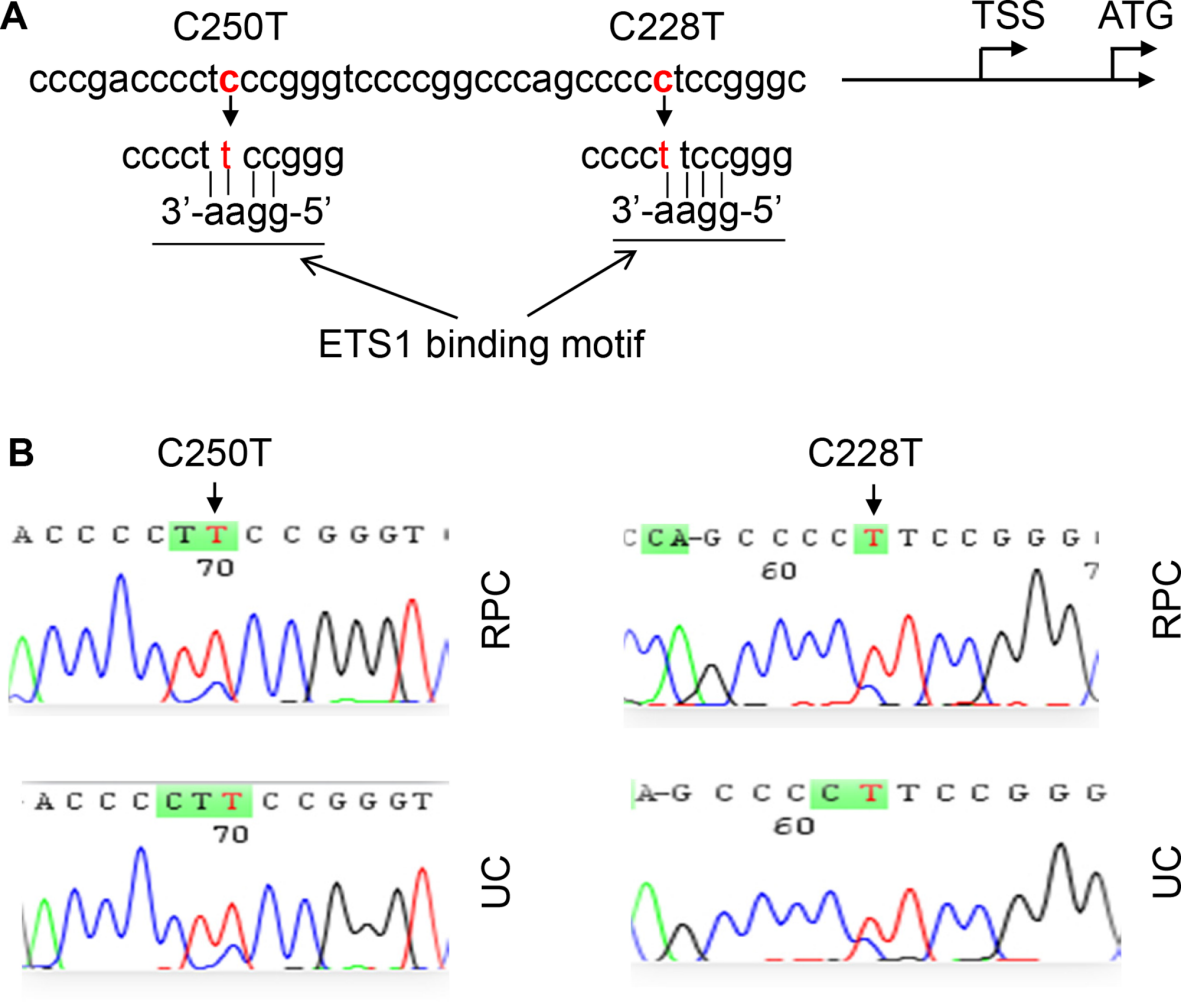
TERT promoter C228T and C250T mutations identified in renal pelvic and ureter carcinomas (RPCs and UCs) **(A)** Location of C228T and C250T (in red) in the TERT core promoter. TSS and ATG: Transcription and translation start sites, respectively. The mutations create *de novo* binding motifs (GGAA) for the transcription factor ETS1. **(B)** Sequencing chromatographs of the TERT promoter locus in tumor genomic DNA from two RPC and 2 UC patients obtained by Sanger sequencing. Left panel: C to T transition at C250. Right panel: C to T transition at C228.

Tumor DNA derived from 220 patients with UTUC was analyzed for the TERT promoter status using Sanger sequencing. A total of 61 patients (27%) were identified to harbor TERT promoter mutations in their tumors (Tables 1 and 2, Fig. 1A and 1B). Forty-two of 98 (43%) RPC tumors carried TERT promoter mutations, in which 38 were C228T and 4 were C250T; while only 23 of 122 (19%) UC tumors had the mutation (15 cases with C228T and 8 with C250T). Hence, TERT promoter mutations occur much more frequently in RPCs than in UCs. C228T mutation was predominant and mutually exclusive with C250T in UTUC tumors.

**Table 1:** Clinical and disease characteristics in relation to *TERT* promoter mutations in patients with renal pelvic carcinoma

	*TERT* promoter mutation		
Variable informative cases (*n* = )	Mutated (*n* = 42)	wild-type (*n* = 56)	*P*-value
*Age (years) at diagnosis* (*n* = 98)			
Mean (± SD)	61.90 ± 10.031	63.96 ± 11.207	n.s. (0.349)
Median (range)	64 (36 − 82)	65.5 (40 − 85)	
*Sex* (*n* = 98)			n.s. (0.646)
Female	14	30	
Male	28	26	
*TNM stage* (*n* = 98)			n.s. (0.216)
pTa + pT1	6	14	
≥ pT2	36	42	
*Pathology stage* (*n* = 98)			n.s. (0.239)
G2	13	11	
G3	29	45	
*Tumor size* (*n* = 93)			n.s. (0.825)
< 3cm	13	16	
≥ 3 cm	27	37	
*Distant metastases* (*n* = 98)			**0.013**
Yes	5	0	
No	37	56	
*Lymph node infiltration* (*n* = 98)			ns.(0.069)
Yes	0	5	
No	42	51	

n.s. = not statistically significant; Significant *P*-values are indicated in bold

**Table 2:** Clinical and disease characteristics in relation to TERT promoter mutations in patients with ureter carcinoma

	*TERT* promoter mutation		
Variable informative cases (*n* = )	Mutated (*n* = 23)	wild-type (*n* = 99)	*P*-value
*Age (years) at diagnosis* (*n* = 122)			
Mean (± SD)	72.39 ± 8.994	65.05 ± 9.612	**0.001**
Median (range)	75(55 − 87)	67 (32 − 87)	
Sex (*n* = 122)			n.s. (0.632)
Female	7	38	
Male	16	61	
*TNM stage* (*n* = 122)			n.s. (0.589)
pTa + pT1	4	25	
≥ pT2	19	74	
*Pathological stage* (*n* = 122)			n.s. (1.000)
G2	6	24	
G3	17	75	
*Tumor size* (*n* = 101)			n.s. (0.623)
< 3cm	10	46	
≥ 3 cm	10	35	
*Distant metastases* (*n* = 122)			**0.046**
Yes	3	2	
No	20	97	
*Lymph node infiltration* (*n* = 122)			ns.(0.686)
Yes	1	9	
No	22	90	

n.s. = not statistically significant; Significant *P*-values are indicated in bold.

### The association of TERT promoter mutations with clinical variables in UTUCs

We then determined a potential relationship between TERT promoter mutations and clinical variables in patients with UTUC. Mutations increased in frequency with age in patients with UCs (*P* = 0.001) but not in RPCs (Tables 1 and 2). The distribution of sex, tumor size, clinical and pathological stages and local lymph node infiltration did not differ significantly between RPC or UC patients with and without TERT promoter mutations. In both RPCs and UCs, however, distant metastasis was closely associated with the presence of TERT promoter mutations: All 4 RPC patients with distant metastasis were mutation-positive whereas none of 56 patients with a wild-type TERT promoter had metastatic disease (*P* = 0.013, Fisher exact test, Table 1). Three of 23 (13%) UC patients who had distant metastasis carried a C228T mutation while this was observed in only 2 of 99 (2%) mutation-negative tumors (*P* = 0.046, Table 2). Altogether, distant metastatic disease occurred in eight of 64 (12.5%) and 2 of 155 (1.3%) patients with tumors carrying and lacking TERT promoter mutations, respectively (*P* = 0.001, *X*^ 2^ test).

### Detection of TERT promoter mutations in urine from patients with UTUC by Sanger sequencing

**Figure 2:**
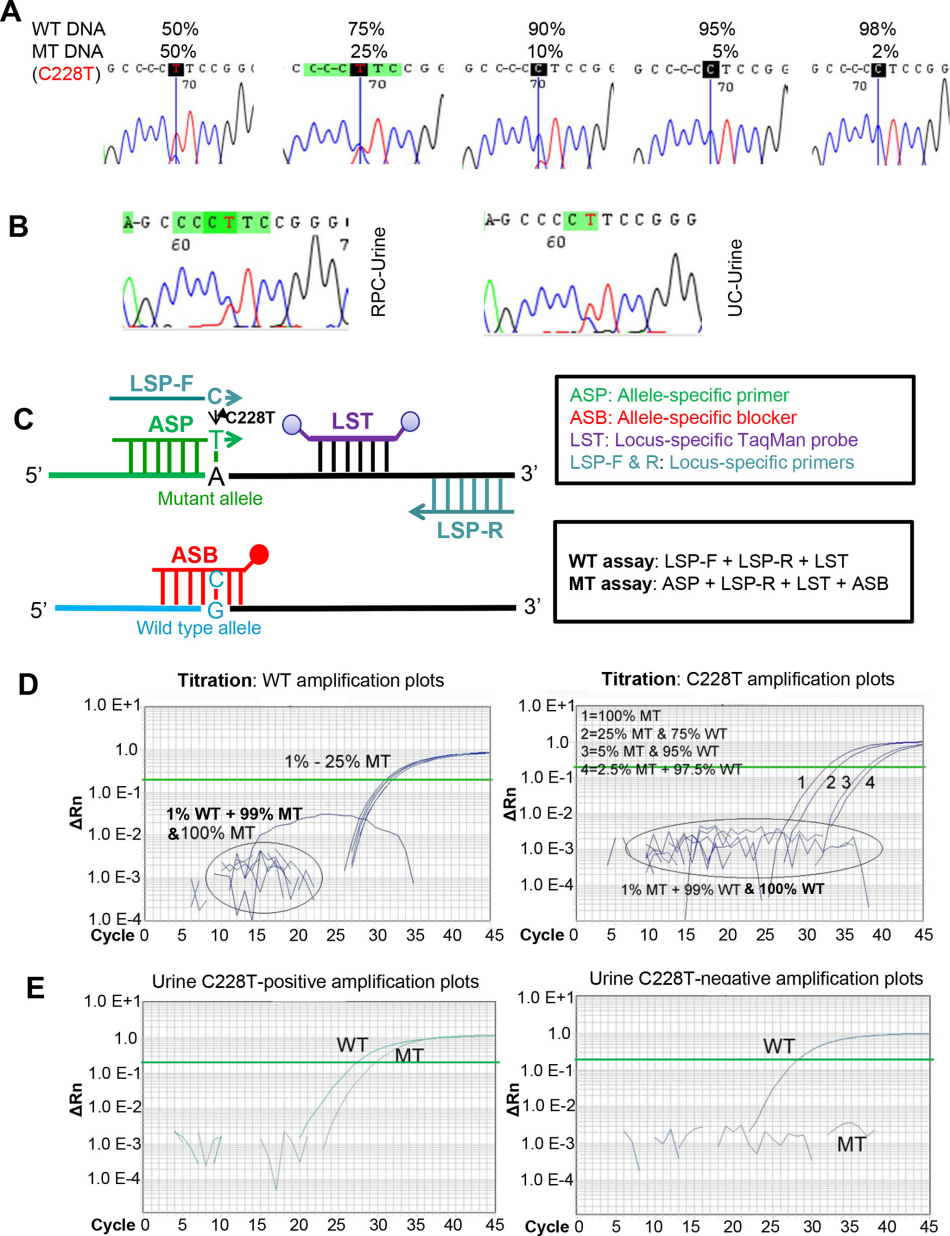
The increased sensitivity for the castPCR detection of C228T mutation in urine derived from patients with renal pelvic and ureter carcinomas (RPCs and UCs) MT and WT, Mutant and wild type TERT promoters, respectively. **(A)** The detection sensitivity of TERT promoter mutations as determined by Sanger sequencing. DNA derived from thyroid cancer cells with (homozygous) C228T mutation and with a wt TERT promoter was mixed as indicated and the promoter region then sequenced using Sanger sequencing. The detectable load of mutant DNA by Sanger sequencing was minimally 10%. **(B)** Sequencing chromatographs of the TERT promoter locus in urine DNA from one renal pelvic carcinoma (RPC) and one ureter carcinoma (UC) patient, as determined by Sanger sequencing. C228T mutation was shown. **(C)** Schematic illustration of the castPCR detection of C228T mutation. The C228T and wt (reference) allele assays are performed with the allele-specific primer (ASP), locus-specific primer, allele-specific blocker (ASB) and locus-specific Taqman probe (LST). In mutant assays, ASB prevents wt TERT promoter sequences from PCR amplification. **(D)** Representative amplification plots for the assay of different proportions of C228T mutant allele as determined by castPCR. Mixed DNA as above in (A) was analyzed for the presence of the C228T mutation using castPCR. Left panel: The amplification plots for the wt (reference) TERT promoter. 99%, 97.5%, 95% and 75% wt DNA-containing mixtures were amplified using **LSP** primers and wild type plots were generated. Of note, pure (100%) and 99% mutant DNA only yielded background signals. Right panel: The amplification plots for the mutant TERT promoter. The same DNA mixtures as described above were amplified using **ASP** primers and mutant amplification plots were generated. The CT value was inversely correlated with % of the mutant alleles. Of note, pure (100%) and 99% wt DNA-containing mixtures only gave rise to background signals. **(E)** C228T mutation-positive (Left) and Negative (Right) urine as revealed by castPCR. Shown are representative castPCR results obtained from two RPC patients' urine samples.

Having identified TERT promoter mutations in RPC and UC tumors, we sought to probe whether they could be detected in the urine of these patients. Urinary DNA from 36 patients with UTUC (16 RPCs and 20 UCs) collected prior to surgical treatment was first analysed (Tables 3 and 4). In 16 RPC patients with urine samples available, 8 had mutation-positive tumors and remaining 8 tumors were wild-type. Sanger sequencing showed that the mutant TERT promoter was detectable in urine samples from 4 of 8 patients with mutation-positive RPC (Table 3, Fig. 2B). Large-sized tumors tended to be more easily detected (5.4 ± 2.3 vs 3.8 ± 2.3), but the difference was not significant (*P* = 0.26) (Table 3). Of note, the mutant TERT sequence was also found in one of 8 urine specimens from 8 cases with mutation-negative RPC. Two of twenty analysed UC tumors carried the C228T mutation, and the same mutation was detected in their urine, too (Table 4, Fig. 2B). The Sanger sequencing result revealed the presence of a wild-type TERT promoter in the remaining 18 urine samples from 18 UC patients with a wild-type TERT promoter. In addition, we also consecutively collected urine samples from 4 RPC and 9 UC patients one week after surgery and all three patients with mutation-positive urine samples prior to operation became negative following surgical resection of tumors (Tables 3 and 4). Altogether, Sanger sequencing yields a 60% sensitivity and 96% specificity for urine detection of TERT promoter mutations in UTUC patients (Table 5).

**Table 3:** TERT promoter mutations detected in both tumor and urine samples from RPC patients

Case number	Sex M / F	Age at diagnosis (year)	Tumor size(CM)	TNM stage	TERT promoter mutation				
					Tissue	Preoperative urine		Postoperative urine	
					Sanger		castPCR	Sanger	castPCR
RPC-1	F	68	3.5	T1N0M0	wt	wt	wt	wt	wt
RPC-2	F	66	3	T1N0M0	wt	wt	wt	NA	NA
RPC-3	F	61	10	T1N0M0	wt	wt	wt	NA	NA
RPC-4	M	71	8	T3N0M0	C228T	C228T	C228T	NA	NA
RPC-5	M	43	5.5	T3N0M0	C228T	wt	C228T	NA	NA
RPC-6	M	65	1.8	T1N0M0	wt	wt	wt	wt	wt
RPC-7	F	82	6	T2N0M0	C228T	C228T	C228T	wt	wt
RPC-8	M	79	1	T1N0M0	wt	wt	C228T	NA	NA
RPC-9	M	71	3.5	T3N0M0	C228T	wt	C228T	NA	NA
RPC-10	M	63	5	T4N0M0	C228T	C228T	C228T	wt	wt
RPC-11	M	64	2.5	T3N0M0	C228T	C228T	C228T	NA	NA
RPC-12	M	64	3	T2N0M0	C228T	wt	wt	NA	NA
RPC-13	F	67	3.2	T1N0M0	C228T	wt	C228T	NA	NA
RPC-14	M	80	4.8	T3N0M0	wt	wt	wt	NA	NA
RPC-15	F	73	4.5	T2N0M0	wt	wt	wt	NA	NA
RPC-16	M	57	2	T3N0M0	wt	C228T	C228T	NA	NA

RPC, Renal pelvic carcinoma; M, Male; F, Female; castPCR, Competitive Allele-specific TaqMan PCR; NA, Not available; wt, Wild type; Sanger, Sanger sequencing

**Table 4:** TERT promoter mutations detected in both tumor and urine samples from UC patients

Case number	Gender M / F	Age at diagnosis (year)	Tumor size(CM)	TNM stage	TERT promoter mutation				
					Tissue	Preoperative urine		Postoperative urine	
					Sanger sequencing		castPCR	Sanger sequencing	castPCR
UC-1	M	67	2.5	T2N0M0	wt	wt	wt	NA	NA
UC-2	F	72	0.3	T1N0M0	wt	wt	wt	NA	NA
UC-3	F	68	1.7	T3N0M0	wt	wt	wt	NA	NA
UC-4	M	71	1.2	TaN0M0	wt	wt	wt	NA	NA
UC-5	M	67	4	T3N0M0	wt	wt	wt	NA	NA
UC-6	M	61	2	T1N0M0	wt	wt	C228T	NA	NA
UC-7	F	68	4.5	T1N0M0	wt	wt	wt	NA	NA
UC-8	M	78	4.5	TaN0M0	C228T	C228T	C228T	NA	NA
UC-9	F	67	1.6	T3N0M0	wt	wt	wt	NA	NA
UC-10	F	54	4	T3N2M1	wt	wt	wt	wt	wt
UC-11	M	65	3.5	T3N0M0	wt	wt	wt	NA	NA
UC-12	M	50	2.2	T2N0M0	wt	wt	wt	wt	wt
UC-13	F	58	1.9	T3N0M0	wt	wt	wt	wt	wt
UC-14	M	49	3	T1N0M0	wt	wt	wt	wt	wt
UC-15	M	70	4	T2N0M1	wt	wt	wt	wt	wt
UC-16	F	65	1.7	T2N0M0	Wt	wt	wt	wt	wt
UC-17	F	61	1.6	T1N0M0	Wt	wt	wt	wt	wt
UC-18	F	61	5	T2N0M0	wt	wt	wt	wt	wt
UC-19	M	57	1.5	T1N0M1	C228T	C228T	C228T	wt	wt
UC-20	M	69	3.7	T3N0M0	wt	wt	wt	NA	NA

UC, Ureter carcinoma; M, Male; F, Female; castPCR, Competitive Allele-specific TaqMan PCR; NA, Not available; Wt, Wild type.

**Table 5:** Concordance of C228T mutation between tumor tissues and urine samples as determined using Sanger sequencing and castPCR

	Tumor tissues					
UTUC	Sanger	Mutant	WT	Sensitivity	Specificity	Accuracy
Urine samples	Mutant	6	1	60% (26 – 88%*)	97% (86 – 100%)	89%
	WT	4	36			
	Total	10	37			

	castPCR	Mutant	WT	Sensitivity	Specificity	Accuracy
Urine samples	Mutant	9	3	90%(56 – 100%)	92% (78 – 98%)	94%
	WT	1	34			
	Total	10	37			

	Tumor tissues					
BC	Sanger	Mutant	WT	Sensitivity	Specificity	Accuracy
Urine samples	Mutant	17	0	47% (30 – 65%)	100% (89 –100%)	73%
	WT	19	33			
	Total	36	33			

	castPCR	Mutant	WT	Sensitivity	Specificity	Accuracy
Urine samples	Mutant	31	1	86% (67 – 94%)	97% (82 – 100%)	90%
	WT	5	32			
	Total	36	33			

	Tumor tissues					
UTUC + BC	Sanger	Mutant	WT	Sensitivity	Specificity	Accuracy
Urine samples	Mutant	23	1	50% (35 – 65%)	98% (92 – 100%)	76%
	WT	23	69			
	Total	46	70			

	castPCR	Mutant	WT	Sensitivity	Specificity	Accuracy
Urine samples	Mutant	40	3	89% (74 – 96%)	96% (88 – 99%)	91%
	WT	6	67			
	Total	46	70			

McNemar's Test for differences between Sanger sequencing and castPCR assays

Sensitivity: UTUC, *P* = 0.37; BC, *P* = 0.003; UTUC +BC: *P* < 0.001.

Specificity: UTUC, BC and UTUC + BC: *P* ≥ 0.48.

*95% confidence intervals.

### castPCR detection of TERT promoter mutations in urine from patients with UTUC

**Figure 3:**
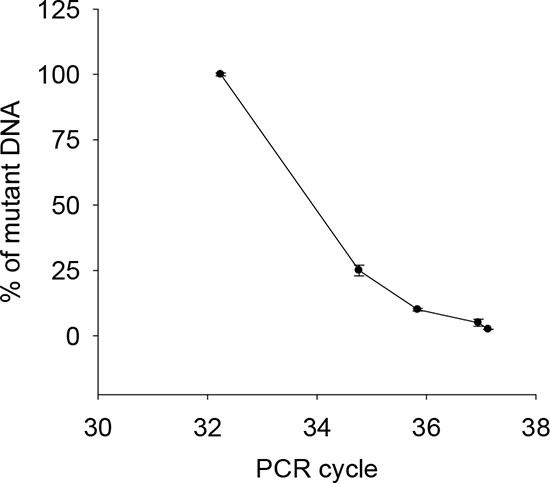
Correlation between the abundance of the mutant C228T and CT values by castPCR Mixed DNA from one C228T-positive (U-hth 7) and one wild-type TERT promoter-carrying (ARO) thyroid cancer line was analyzed using castPCR. The percentage of C228T was in general correlated with CT values. The threshold abundance was 2.5% of the mutant. The result was based on three independent assays. Bars: SD.

To potentially improve the capacity to detect TERT promoter mutations in the urine from UTUC patients, we applied castPCR, a sensitive PCR-based technique for a low abundance of mutant genes [22]. To do this, we first compared the sensitivity and specificity of Sanger sequencing and castPCR by using mixed DNA from one C228T-positive and one wild-type TERT promoter-carrying thyroid cancer line. The threshold limit for castPCR and Sanger sequencing detection was 2.5% and 10% of mutant alleles present in mixed DNA samples, respectively (Fig. 2A, 2C and 2D, and Fig. 3), which indicate at least four-fold higher detection sensitivity by castPCR than by Sanger sequencing (Fig. 2A and 2D). Thus, we then employed castPCR to determine TERT promoter mutations in urine samples from 36 patients as described above (Tables 3 and 4). The castPCR analysis showed the presence of C228T mutations in 9 of 10 urine samples from patients with C228T-positive tumors prior to operation (Tables 3 and 4, Fig. 2E). Among these 9 castPCR C228T positive urine samples, 3 were undetectable using Sanger sequencing. Thus, the assay sensitivity for castPCR reached 90%, substantially higher than the recorded 60% using Sanger sequencing. Thirty-four of 37 urine samples collected before and after surgery from 26 patients with tumors harbouring a wild-type TERT promoter were documented as mutant-negative by castPCR. The sensitivity and specificity of the castPCR assay in this serial of patients were 90% and 92%, respectively (Table 5).

### The verification of the sensitivity and specificity for the castPCR assay in urine from BC patients

A high sensitivity and specificity of urinary detection of C228T mutation by castPCR, as seen above, was based on the analysis of only 36 UTUC patients, and further verification of the castPCR assay was therefore required. For this purpose, we examined 70 urine and tumor samples obtained from 70 patients with BC at diagnosis. The patient clinical features were provided in Supplementary Table S1. The Sanger sequencing revealed that 41 of 70 BC tumors harboured TERT promoter mutations. In these 41 mutation-carrying tumors, 36 were C228T mutation and Sanger sequencing of urinary DNA samples from these 36 patients demonstrated that 17 of them (17/36, 47%) contained C228T mutant, too (Supplementary Table S1). The castPCR assay showed that 31 of these 36 urine specimens (86%) were positive for C228T mutation (Sanger vs castPCR, *P* = 0.003, Table 5). Both Sanger sequencing and castPCR assays were highly specific (Table 5).

Taken together, both sets of urine analyses of UTUC and BC patients demonstrate that the castPCR detection of urinary C228T mutation significantly increased sensitivity and accuracy without compromising specificity. The overall sensitivity was 89% (75 – 96%, 95% CI) and 50% (35 – 65%) for castPCR and Sanger sequencing (*P* < 0.001), while their specificity was 97% and 98%, respectively (*P* = 0.48) (Table 5).

## DISCUSSION

The identification of recurrent TERT promoter mutations in human malignancies has significantly contributed to the understanding of the cancer-specific TERT expression. Biologically, the finding of TERT promoter mutations reveals a novel mechanism activating telomerase in oncogenesis. Mutation create *de novo* ETS binding motifs and facilitates TERT transcription, thereby leading to telomerase activation [6, 10]. Clinically, the mutation is widespread in certain types of cancer, and likely serves as a useful diagnostic and/or prognostic marker [7–9].

The present study shows a high rate of TERT promoter mutations in UTUC tumors, particularly in RPCs where the mutation frequency reached 43%. To our knowledge, TERT appears to be the most commonly mutated gene ever reported in RPCs. The presence of TERT promoter mutations was closely associated with distant metastases in both RPCs and UCs. Moreover, the mutant sequence is detectable in patients' urine. Collectively, our findings indicate that the TERT promoter mutation assay has potentially important clinical implication in UTUCs.

Both UTUCs and BCs originate from the urothelium and belong to transitional cell carcinomas [19, 20]. They also share common genetic alterations, such as the mutation of *fibroblast growth factor receptor 3 (FGFR3)* [23]. It was shown that the selective TERT promoter mutational profile was associated with tumor histology and tissue of origin [7, 12], and it is thus surprising to observe a low frequency of TERT promoter mutations in UCs. Up to 84% of BC tumors carry TERT promoter mutations [11], which is four-fold higher than that observed in UC tumors (Fig. S1). Further investigations are required to elucidate the mechanism behind differential TERT promoter mutations between these two closely related malignancies with the same origin.

The striking correlation between TERT promoter mutations and distant metastases in UTUCs suggests a role of TERT in UTUC dissemination. Likely, the promoter mutation-induced TERT expression provides cancer cells with a proliferation advantage by stabilizing telomere size. Wu et al [10] showed that BC cells with either C228T or C250T mutations acquired enhanced cellular motility. In addition, TERT displays multiple activities independently of telomere lengthening and is capable of protecting cancer cells from apoptosis stimulated by various insults or stresses [24–30]. More importantly, TERT could directly promote cancer cell invasion and metastasis by inducing epithelial-mesenchymal transition [31]. Taken together, the TERT promoter mutation-mediated TERT expression may contribute to remote metastases of cancer cells via both telomere lengthening-dependent and independent mechanisms.

Urine-based tests are a non-invasive diagnostic tool for urological malignancies including BC and UTUC [7, 8, 19]. Since normal human cells lack TERT promoter mutations, they are likely ideal urinary markers for diagnosis and disease monitoring of these urological cancers [7–9]. Direct sequencing such as Sanger sequencing is regarded as a gold standard for the identification of mutant targets, however, voided urine contains both normal and malignant cells, and if the latter fraction is too small, it might be difficult to catch the mutant target by Sanger sequencing. Such a scenario is expected with small either primary or recurring tumors producing few exfoliated tumor cells. Thus, this motivated us to develop more sensitive assays to detect minor proportions of mutant alleles present in bulk urinary DNA. Based on the cell line titration test, the threshold sensitivity of Sanger sequencing is at least 10% of mutant TERT promoter-containing tumor DNA while castPCR could increase the detection limit to 2.5%. Consistent with the titration result, patients' urine tests showed 60% and 90% of detection sensitivity with Sanger sequencing and castPCR, respectively. However, castPCR yielded 92% specificity, slightly lower than Sanger sequencing (97%). Similar results were also obtained from the analysis of 70 BC urine specimens. Further optimization of the castPCR assay will improve its detection accuracy.

In summary, TERT promoter mutations occurred in 43% and 19% of RPC and UC tumors, respectively. The presence of TERT promoter mutations predicts metastatic disease in UTUCs. The castPCR assay exhibited a high sensitivity and specificity for detection of the mutant TERT promoter in urine from RPC and BC patients. These results may have important clinical implications in the diagnostics and management of patients with urological malignancies.

## MATERIALS AND METHODS

### Patients and tumor specimens

The study was conducted on 98 patients with RPC and 122 patients with UC who underwent surgery at Shandong University Qilu Hospital and Second Hospital, China. RPC and UC were diagnosed according to the criteria of the World Health Organization [32]. Patients' characteristics are summarized in Tables 1 and 2. The specimens were collected after surgical treatment and kept frozen at −70 °C or paraffin-embedded until use. All samples were collected with written informed consent and the study was approved by the Institutional research ethics committee.

### Voided urine samples from patients with RPC and UC

Spontaneously voided urine was collected from 16 RPC and 20 UC patients prior to surgical treatment. In 13 of these 36 patients, urine was also consecutively obtained one week post-operation. Fifty ml of urine were centrifuged and cell pellets were kept at −70 °C until use.

### DNA extraction and sequencing

Genomic DNA was extracted from frozen and/or paraffin-embedded tumor samples and urine pellets using QIAGEN DNA extraction kits. The two hotspot mutations defined as C228T and C250T in the TERT core promoter correspond to positions 124 and 146 bp upstream of the ATG site (Fig. 1A). The target region covering these two hotspots were amplified using conventional PCR followed by Sanger sequencing as described [18, 31]. The PCR was performed with the following primer pairs: 5′-CACCCGTCCTGCCCCTTCACCTT-3′ and 5′- GGCTTCCCACGTGCGCAGCAGGA-3′. The mutations were verified by sequencing from both directions.

### castPCR

castPCR analysis was performed by using ABI 7900 Real-time PCR system. Ten μl of PCR reaction volume in 384 wells plate included 5 μl of 2 × TaqMan genotyping master mixture, 1 μl of 10 × Assay Mixture buffer (mutant or wt), 2 μl H_2_O and 20 ng DNA template (diluted to 10ng/μl). Thermo cycling conditions were: 95°C for 10 mins, then (92°C for 15s and 58°C for 1 min) × 5 cycles followed by additional 45 cycles with 92°C for 15s and 60°C for 1 min. The PCR result was analyzed with the SDS 2.4 software program and Mutation Detector Software 2.0 (Life Technologies) (http://tools.lifetechnologies.com/content/sfs/brochures/cms_095916.pdf.). To test the sensitivity and specificity of the mutant TERT promoter detection by castPCR, urinary DNA from UTUC patients was analyzed for C228T mutation using castPCR and Sanger sequencing, and the obtained results were then compared based on the TERT promoter status in tumors. In addition, the sensitivity and specificity of the castPCR assay was further evaluated by assessing urine and tumor specimens derived from 70 patients with BC.

### Statistical analyses

Differences in the TERT promoter mutation frequency in relation to sex, clinical and pathological stage were determined using Fisher's exact or *X*^2^ test. Student's T-test was used to analyze differences in age and tumor size between the TERT promoter mutation-positive and negative groups, respectively. Sensitivity and specificity differences between castPCR and Sanger sequencing assays were evaluated using McNemar's Test. All the tests were two-tailed and computed using SigmaStat3.1® software (Systat Software, Inc., Richmond, CA). *P* values of < 0.05 were considered as statistically significant.

## SUPPLEMENTARY TABLE AND FIGURE



## References

[1] Daniel M, Peek GW, Tollefsbol TO (2012). Regulation of the human catalytic subunit of telomerase (hTERT). Gene.

[2] Shay JW, Wright WE (2011). Role of telomeres and telomerase in cancer. Semin Cancer Biol.

[3] Kong F, Zheng C, Xu D (2014). Telomerase as a “stemness” enzyme. Sci China Life Sci.

[4] Heidenreich B, Rachakonda PS, Hemminki K, Kumar R (2014). TERT promoter mutations in cancer development. Curr Opin Genet Dev.

[5] Horn S, Figl A, Rachakonda PS, Fischer C, Sucker A, Gast A, Kadel S, Moll I, Nagore E, Hemminki K, Schadendorf D, Kumar R (2013). TERT promoter mutations in familial and sporadic melanoma. Science.

[6] Huang FW, Hodis E, Xu MJ, Kryukov GV, Chin L, Garraway LA (2013). Highly recurrent TERT promoter mutations in human melanoma. Science.

[7] Allory Y, Beukers W, Sagrera A, Flandez M, Marques M, Marquez M, van der Keur KA, Dyrskjot L, Lurkin I, Vermeij M (2014). Telomerase Reverse Transcriptase Promoter Mutations in Bladder Cancer: High Frequency Across Stages, Detection in Urine, and Lack of Association with Outcome. Eur Urol.

[8] Hurst CD, Platt FM, Knowles MA (2014). Comprehensive mutation analysis of the TERT promoter in bladder cancer and detection of mutations in voided urine. Eur Urol.

[9] Wang K, Liu T, Liu L, Liu J, Liu C, Wang C, Ge N, Ren H, Yan K, Hu S, Björkholm, Fan Y, Xu D (2014). TERT promoter mutations in renal cell carcinomas and upper tract urothelial carcinomas. Oncotarget.

[10] Wu S, Huang P, Li C, Huang Y, Li X, Wang Y, Chen C, Lv Z, Tang A, Sun X (2014). Telomerase reverse transcriptase gene promoter mutations help discern the origin of urogenital tumors: a genomic and molecular study. Eur Urol.

[11] Liu X, Wu G, Hartmann C, Xing M (2013). Highly prevalent TERT promoter mutations in bladder cancer and brain gliobastoma. Cell Cycle.

[12] Killela PJ, Reitman ZJ, Jiao Y, Bettegowda C, Agrawal N, Diaz LA, Friedman AH, Friedman H, Gallia GL, Giovanella BC, Grollman AP, He TC, He Y, Hruban RH, Jallo GI, Mandahl N, Meeker AK, Mertens F, Netto GJ, Rasheed BA, Riggins GJ, Rosenquist TA, Schiffman M, Shih IeM, Theodorescu D, Torbenson MS, Velculescu VE, Wang TL, Wentzensen N, Wood LD, Zhang M, McLendon RE, Bigner DD, Kinzler KW, Vogelstein B, Papadopoulos N, Yan H (2013). TERT promoter mutations occur frequently in gliomas and a subset of tumors derived from cells with low rates of self-renewal. Proc Natl Acad Sci U S A.

[13] Rachakonda PS, Hosen I, de Verdier PJ, Fallah M, Heidenreich B, Ryk C, Wiklund NP, Steineck G, Schadendorf D, Hemminki K, Kumar R (2013). TERT promoter mutations in bladder cancer affect patient survival and disease recurrence through modification by a common polymorphism. Proc Natl Acad Sci U S A.

[14] Tallet A, Nault JC, Renier A, Hysi I, Galateau-Salle F, Cazes A, Copin MC, Hofman P, Andujar P, Le Pimpec-Barthes F (2014). Overexpression and promoter mutation of the TERT gene in malignant pleural mesothelioma. Oncogene.

[15] Killela PJ, Pirozzi CJ, Healy P, Reitman ZJ, Lipp E, Rasheed BA, Yang R, Diplas BH, Wang Z, Greer PK, Zhu H, Wang CY, Carpenter AB, Friedman H, Friedman AH, Keir ST, He J, He Y, McLendon RE, Herndon JE, Yan H, Bigner DD (2014). Mutations in IDH1, IDH2, and in the TERT promoter define clinically distinct subgroups of adult malignant gliomas. Oncotarget.

[16] Xie H, Liu T, Wang N, Björnhagen V, Höög A, Larsson C, Lui WO, Xu D (2014). TERT promoter mutations and gene amplification: Promoting TERT expression in Merkel cell carcinoma. Oncotarget.

[17] Liu T, Wang N, Cao J, Dinets A, Sofiadis A, Zedenius J, Larsson C, Xu D (2014). The age- and shorter telomere-dependent TERT promoter mutation in follicular thyroid cell-derived carcinomas. Oncogene.

[18] Wang N, Liu T, Sofiadis A, Juhlin CC, Zedenius J, Hoog A, Larsson C, Xu D (2014). TERT promoter mutation as an early genetic event activating telomerase in follicular thyroid adenoma (FTA) and atypical FTA. Cancer.

[19] Roupret M, Babjuk M, Comperat E, Zigeuner R, Sylvester R, Burger M, Cowan N, Bohle A, Van Rhijn BW, Kaasinen E (2013). European guidelines on upper tract urothelial carcinomas: 2013 update. Eur Urol.

[20] Chromecki TF, Bensalah K, Remzi M, Verhoest G, Cha EK, Scherr DS, Novara G, Karakiewicz PI, Shariat SF (2011). Prognostic factors for upper urinary tract urothelial carcinoma. Nat Rev Urol.

[21] Nakanishi K, Hiroi S, Kawai T, Aida S, Kasamatsu H, Aurues T, Ikeda T (2001). Expression of telomerase catalytic subunit (hTERT) mRNA does not predict survival in patients with transitional cell carcinoma of the upper urinary tract. Mod Pathol.

[22] Thierry AR, Mouliere F, El Messaoudi S, Mollevi C, Lopez-Crapez E, Rolet F, Gillet B, Gongora C, Dechelotte P, Robert B (2014). Clinical validation of the detection of KRAS and BRAF mutations from circulating tumor DNA. Nat Med.

[23] van Oers JM, Zwarthoff EC, Rehman I, Azzouzi AR, Cussenot O, Meuth M, Hamdy FC, Catto JW (2009). FGFR3 mutations indicate better survival in invasive upper urinary tract and bladder tumours. Eur Urol.

[24] Cao Y, Li H, Deb S, Liu JP (2002). TERT regulates cell survival independent of telomerase enzymatic activity. Oncogene.

[25] Cong Y, Shay JW (2008). Actions of human telomerase beyond telomeres. Cell Res.

[26] Ding D, Xi P, Zhou J, Wang M, Cong YS (2013). Human telomerase reverse transcriptase regulates MMP expression independently of telomerase activity via NF-kappaB-dependent transcription. Faseb J.

[27] Ghosh A, Saginc G, Leow SC, Khattar E, Shin EM, Yan TD, Wong M, Zhang Z, Li G, Sung WK (2012). Telomerase directly regulates NF-kappaB-dependent transcription. Nat Cell Biol.

[28] Luiten RM, Pene J, Yssel H, Spits H (2003). Ectopic hTERT expression extends the life span of human CD4+ helper and regulatory T-cell clones and confers resistance to oxidative stress-induced apoptosis. Blood.

[29] Saretzki G (2014). Extra-telomeric Functions of Human Telomerase: Cancer, Mitochondria and Oxidative Stress. Curr Pharm Des.

[30] Ahmed S, Passos JF, Birket MJ, Beckmann T, Brings S, Peters H, Birch-Machin MA, von Zglinicki T, Saretzki G (2008). Telomerase does not counteract telomere shortening but protects mitochondrial function under oxidative stress. J Cell Sci.

[31] Liu Z, Li Q, Li K, Chen L, Li W, Hou M, Liu T, Yang J, Lindvall C, Bjorkholm M, Jia J, Xu D (2013). Telomerase reverse transcriptase promotes epithelial-mesenchymal transition and stem cell-like traits in cancer cells. Oncogene.

[32] Lopez-Beltran A, Bassi P, Pavone-Macaluso M, Montironi R (2004). Handling and pathology reporting of specimens with carcinoma of the urinary bladder, ureter, and renal pelvis. Eur Urol.

