# Supplementary material for: TERT promoter mutations are associated with distant metastases in upper tract urothelial carcinomas and serve as urinary biomarkers detected by a sensitive castPCR
Source: Oncotarget. 2014 Dec 9;5(23):12428–39. doi: 10.18632/oncotarget.2660 (PMC4322995; doi:10.18632/oncotarget.2660)
Supplement: Supplementary file 1 [file oncotarget-05-12428-s001.pdf]

SUPPLEMENTARY TABLE AND FIGURE

Supplementary Table S1. TERT promoter mutations in urine samples from patients with bladder cancer detected using Sanger sequencing and castPCR.

Case number	Gender M / F	Age at diagnosis (year)	Tumor size (CM)	TNM stage	TERT promoter mutation		
					Tissue	Urine	
					Sanger squencing		castPCR
BC-1	M	70	3	TaN0M0	C228T	C228T	C228T
BC-2	M	71	3.2	T1N0M0	C228T	C228T	C228T
BC-3	M	68	3	T2bN0M0	C228T	C228T	C228T
BC-4	M	64	4	T1N0M0	C228T	C228T	C228T
BC-5	M	60	2	T1N0M0	C228T	C228T	C228T
BC-6	M	75	1.5	TaN0M0	C228T	C228T	C228T
BC-7	F	70	3	TaN0M0	C228T	C228T	C228T
BC-8	F	65	2	T3aN0M0	C228T	C228T	C228T
BC-9	M	73	1.5	T1N0M0	C228T	C228T	C228T
BC-10	F	60	1.9	TaN0M0	C228T	C228T	C228T
BC-11	M	55	8.1	T1N0M0	C228T	C228T	C228T
BC-12	M	57	2.1	T1N0M0	C228T	C228T	C228T
BC-13	M	56	3.7	T1N0M0	C228T	C228T	C228T
BC-14	M	74	NA	T1N0M0	C228T	C228T	C228T
BC-15	M	69	4	T2bN0M0	C228T	C228T	C228T
BC-16	M	70	2	T1N0M0	C228T	C228T	C228T
BC-17	M	51	2.7	T3bN2M0	C228T	C228T	C228T
BC-18	M	89	NA	T1N0M0	C228T	WT	WT
BC-19	M	63	NA	T1N0M0	C228T	WT	C228T
BC-20	M	47	2.5	TaN0M0	C228T	WT	C228T
BC-21	M	63	2	TaN0M0	C228T	WT	WT
BC-22	M	78	3	TaN0M0	C228T	WT	WT
BC-23	F	70	3.3	T3aN0M0	C228T	WT	C228T
BC-24	M	71	2.5	T1N0M0	C228T	WT	C228T
BC-25	M	59	7	T3aN0M0	C228T	WT	C228T
BC-26	M	46	1	TaN0M0	C228T	WT	C228T
BC-27	M	44	1	TaN0M0	C228T	WT	C228T
BC-28	M	71	1.5	T1N0M0	C228T	WT	C228T
BC-29	M	59	NA	TisN0M0	C228T	WT	C228T
BC-30	M	58	1.6	T1N0M0	C228T	WT	C228T
BC-31	M	64	0.5	TaN0M0	C228T	WT	WT
BC-32	M	82	1.5	TaN0M0	C228T	WT	WT
BC-33	M	59	5	T3aN0M0	C228T	WT	C228T
BC-34	M	60	2.7	TaN0M0	C228T	WT	C228T

(*Continued*)

Case number	Gender M / F	Age at diagnosis (year)	Tumor size (CM)	TNM stage	TERT promoter mutation		
					Tissue	Urine	
					Sanger squencing		castPCR
BC-35	M	53	7	T2aN0M0	C228T	WT	C228T
BC-36	M	55	2.2	TaN0M0	C228T	WT	C228T
BC-38	F	54	4	T3aN2M0	WT	WT	WT
BC-39	F	83	3.1	TaN0M0	WT	WT	WT
BC-40	M	48	4.1	TaN0M0	WT	WT	WT
BC-41	M	66	2	TaN0M0	WT	WT	WT
BC-42	M	65	NA	T4N0M0	WT	WT	WT
BC-43	M	66	6	T3aN0M0	WT	WT	WT
BC-44	F	79	1	TaN0M0	WT	WT	WT
BC-45	M	67	2.1	TaN0M0	WT	WT	WT
BC-46	M	60	1.5	TaN0M0	WT	WT	C228T
BC-47	M	70	4	T1N0MO	WT	WT	WT
BC-48	M	48	3.2	TaN0M0	WT	WT	WT
BC-49	M	57	2.8	T1N0M0	WT	WT	WT
BC-50	M	75	1	TaN0M0	WT	WT	WT
BC-51	M	73	2	TaN0M0	WT	WT	WT
BC-52	M	66	4.3	T1N0M0	WT	WT	C228T
BC-53	F	70	8	T3aN0M0	WT	WT	WT
BC-54	F	43	NA	T1N0M0	WT	WT	WT
BC-55	M	73	0.5	TaN0M0	WT	WT	WT
BC-56	F	70	3	T1N0M0	WT	WT	WT
BC-57	M	73	4	T1N0M0	WT	WT	WT
BC-58	M	61	3	TaN0M0	WT	WT	WT
BC-59	M	60	2	TaN0M0	WT	WT	WT
BC-60	F	78	6.5	TaN0M0	WT	WT	WT
BC-61	M	60	8.2	T3bN0M0	WT	WT	WT
BC-62	M	81	1.5	T1N0M0	WT	WT	WT
BC-63	M	53	NA	T1N0M0	WT	WT	WT
BC-64	M	69	3.6	T2aN0M0	WT	WT	WT
BC-65	M	70	3	T1N0M0	WT	WT	WT
BC-66	M	50	3	TaN0M0	C228A	C228A	WT
BC-67	M	73	0.8	TaN0M0	C250T	C250T	WT
BC-68	M	58	4	T3aN0M0	C250T	C250T	WT
BC-69	M	47	4	T1N0M0	CC242/3TT	CC242/3TT	WT
BC-70	M	65	1	T1N0M0	C250T	WT	WT

BC, Bladder cancer; M, Male; F, Female; castPCR, Competitive Allele-specific TaqMan PCR; NA, Not available; WT, Wildtype.

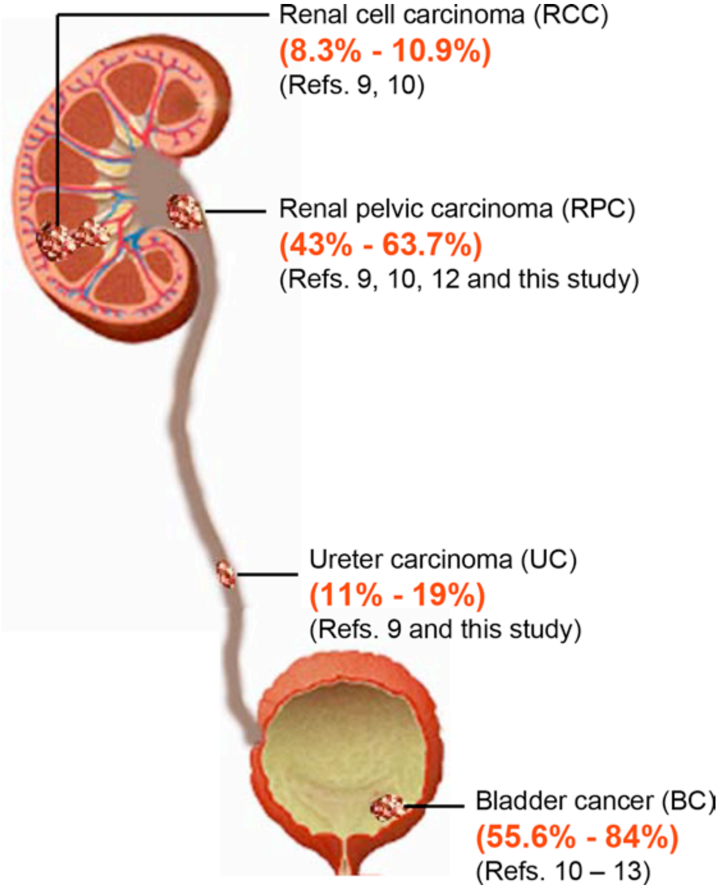

Supplementary Figure S1: Differential frequencies of TERT promoter mutations in urological malignancies. The percentages of the mutation (Red) in each type of cancer are based on the published data and the present study, which exhibit BC>RPC>UC>RCC.
